# Reproductiveaxis gene regulation during photostimulation and photorefractoriness in Yangzhou goose ganders

**DOI:** 10.1186/s12983-017-0200-6

**Published:** 2017-02-23

**Authors:** Huanxi Zhu, Zhe Chen, Xibin Shao, Jianning Yu, Chuankun Wei, Zichun Dai, Zhendan Shi

**Affiliations:** 1 Laboratory of Animal Improvement and Reproduction, Institute of Animal Science, Jiangsu Academy of Agricultural Sciences, Nanjing, 210014 China; 2 Sunlake Swan Farm, Changzhou, 213101 China

**Keywords:** Yangzhou goose ganders, Reproductive activities, Photoperiod, Gene mRNA expressions

## Abstract

**Background:**

The Yangzhou goose is a long-day breeding bird that has been increasingly produced in China. Artificial lighting programs are used for controlling its reproductive activities. This study investigated the regulations of photostimulation and photorefractoriness that govern the onset and cessation of the breeding period.

**Results:**

Increasing the daily photoperiod from 8 to 12 h rapidly stimulated testis development and increased plasma testosterone concentrations, with peak levels being reached 2 months after the photoperiod increase. Subsequently, testicular activities, testicular weight, spermatogenesis, and plasma testosterone concentrations declined steadily and reached to the nadir at 5 months after the 12-hour photoperiod. Throughout the experiment, plasma concentrations of triiodothyronine and thyroxine changed in reciprocal fashions to that of testosterone. The stimulation of reproductive activities caused by the increasing photoperiod was associated with increases in gonadotropin-releasing hormone (GnRH), but decreases in gonadotropin-inhibitory hormone (GnIH) and vasoactive intestinal peptide (VIP) gene messenger RNA (mRNA) levels in the hypothalamus. In the pituitary gland, the levels of follicle-stimulating hormone (FSH) and luteinizing hormone (LH) mRNA abruptly increased during the longer 12-hour photoperiod. The occurrence of photorefractoriness was associated with increased GnIH gene transcription by over 250-fold, together with increased VIP mRNA levels in the hypothalamus, and then prolactin and thyroid-stimulating hormone in the pituitary gland. FSH receptor, LH receptor, and StAR mRNA levels in the testis changed in ways paralleling those of testicular weight and testosterone concentrations.

**Conclusions:**

The seasonal reproductive activities in Yangzhou geese were directly stimulated by a long photoperiod via upregulation of GnRH gene transcription, downregulation of GnIH, VIP gene transcription, and stimulation of gonadotrophin. Development of photorefractoriness was characterized by hyper-regulation of GnIH gene transcription in the hypothalamus, in addition of upregulation of VIP and TRH gene transcription, and that of their receptors, in the pituitary gland.

## Background

Most birds exhibit well-defined seasonal changes in gonadal development, body mass, molting, metabolism, and other physiological parameters [1]. Such seasonal physiological changes are a means of coping with the seasonal fluctuations of environmental factors, such as temperature and food availability, in order to improve survival capacity [2–7]. Seasonality of reproductive activity and that of other physiologic processes in the majority of birds and mammals is presumed to arise from an interaction between endogenous circannual rhythms and a variety of environmental changes, the most important of which is the daily photoperiod [8].

The classic theory of photoperiodic regulation of seasonal reproductive activities in birds proposes that light signals are perceived by photoreceptors in the deep brain, and induce the secretion of thyroid-stimulating hormone (TSH) from the pars tuberalis, which then acts on ependymal cells to induce the thyroid hormone-activating enzyme type 2 deiodinase. This enzyme catalyzes the conversion of thyroxine (T4) to triiodothyronine (T3) [9], which initiates the nervous impulses that lead to the synthesis and release of gonadotropin-releasing hormone (GnRH) [10, 11]. GnRH is then transported by portal blood circulation to the anterior pituitary gland, where it stimulates the synthesis and release of the gonadotropins, luteinizing hormone (LH) and follicle-stimulating hormone (FSH) [12–14]. LH and FSH are responsible for the stimulation of gonad growth and development, as well as for the production of sex steroid hormones [15–18]. The photoperiodic regulation of the annual reproductive cycle requires two kinds of physiologic responses, namely photostimulation and photorefractoriness. The former leads to the activation of the reproductive system and brings animals into the breeding season, and the latter inhibits reproductive activities, terminating the breeding season [3, 14]. In addition to positive regulation by GnRH, the reproductive system is negatively regulated by gonadotropin-inhibitory hormone (GnIH), whose secretion is also subject to photoperiodic regulation [13, 19, 20].

GnIH, which can inhibit LH secretion [20, 21], is produced from the hypothalamic paraventricular nucleus (birds) [22–24] or the dorsomedial nucleus of the hypothalamus (mammals) [25], and is contained in nerve fibers extending to various brain regions, including the pre-optic area and the median eminence, where GnRH perikarya are located. Prolactin (PRL) and its releasing hormone, vasoactive intestinal peptide (VIP), which is secreted by the hypothalamus, are also involved in the regulation of seasonal reproductive activities [26]. For example, the secretion of VIP and PRL, as well as their gene expression, are highly responsive to increases in photoperiod [27–29], and peak PRL concentrations in blood coincide with the onset of gonadal regression [14, 30, 31]. Furthermore, the long photoperiod regulated testicular regression, which was severely retarded, while molting of feathers was blocked in European starlings actively immunized against VIP, which inhibited pituitary PRL secretion [32].

Moreover, it is well established that thyroid hormones play an important role in the regulation of seasonal breeding and other physiological activities such as growth and molting [33, 34]. In many seasonal breeding animals and birds, there is a reciprocal relationship between the plasma concentrations of thyroid hormones and testosterone [34–38]. Based on previous findings showing that thyroidectomy could prevent the development of photorefractoriness in both avian and mammalian species [34, 39, 40], recent investigations in Japanese quail (*Coturnix japonica*) showed that light-induced thyroid hormone synthesis in the mediobasal hypothalamus (MBH) is responsible for the regulation of the neuroendocrine axis involved in seasonal reproduction [41, 42].

**Fig. 1:**
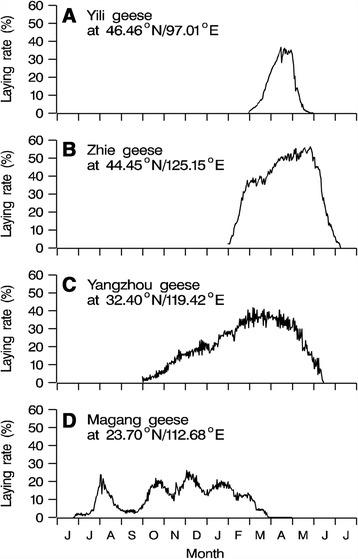
Representative laying patterns of four domestic geese breeds of different reproductive seasonality. Type 1 (**a**) laying in the long-day seasons of March to May (from J.H. Li, unpublished laying record of a flock of 500 geese). Type 2 (**b**) lays entirely during the long-day seasons of spring and early summer (from W. Wu, unpublished laying record of a flock of 650 geese). Type 3 (**c**) starts laying during autumn, but peaks in spring and ends in early summer (from Z.D. Shi, unpublished laying record of a flock of 520 geese). Type 4 (**d**) starts laying in summer when the day length shortens, and ends in the following spring after spring equinox when daily photoperiod increases (from Z.D. Shi, unpublished laying record of a flock of 850 geese)

The thirty some Chinese geese (*Anser cygnoides*) breeds throughout the country all exhibit strong seasonality in breeding activities, despite the fact that they have been domesticated for more than six thousand years [43]. Although these breeds are considered to be of the same genetic origin, as suggested by mitochondrial DNA typing [44], they exhibit divergent breeding seasonality, depending on the geographical location of their habitat. For example, northern breeds are typically long-day breeding birds, whereas the southern breeds are short-day types (Fig. 1) [45]. Such diverse breeding seasonality makes the Chinese geese breeds good model fowls for studying the photoperiodic regulation of seasonal reproduction. Yangzhou goose, a synthetic breed that is widely produced in China, is a long-day breeding fowl, whose egg laying activity starts in autumn when the daily photoperiod decreases, peaks between February and March when the photoperiod lengthens, and ends between May and June when the daily photoperiod further increases and becomes greater than 14 to 16 h [45]. The reproductive seasonality of the Yangzhou goose can be regulated by an artificial photoperiod, and this has been used to induce out-of-season breeding in order to improve economic efficiency of production. In spite of this, the mechanisms of development of photosensitivity and photorefractoriness, which coordinate the formation and length of the reproductive state and are therefore important for breeding and economic efficiency, remain unknown, as do the endocrine, neuroendocrine, and molecular mechanisms that underlie the transduction of photoperiodic signals during the profound fluctuations in reproductive activities. Previous studies and information are scarce, but a preliminary study [46] showed that a long photoperiod of 14 h (14 L:10D) rapidly induced photorefractoriness, and caused reproductive activity to cease not long after it was stimulated. Therefore, in our study, we used the equatorial 12L:12D photoperiod to induce and to maintain reproductive activity. However, to further test the effects of a short-to-long, and a-long-to-longer photoperiod, we designed different photoprotocols in two experimental groups.

This study was designed to unravel the mechanisms of photostimulation and photorefractoriness by investigating the changes in the concentration of plasma testosterone and thyroid hormones, the artificial photoprogram-induced waxing and waning of the testes, and the transcription patterns of relevant genes in the hypothalamus, pituitary gland, and testes.

## Methods

### Experimental design and animals

The experiments were carried out on Sunlake Swan Farm (119°58′E, 31°48′N), Henglin Township, Changzhou, Jiangsu Province, China. Two mechanically ventilated goose barns with an automatic lighting program were used to host the ganders used in the study. On March 1, 2015, a flock of Yangzhou goose ganders (*n* = 210) of 112 days of age and the same genetic origin were equally divided into two groups: A and B.

**Fig. 2:**
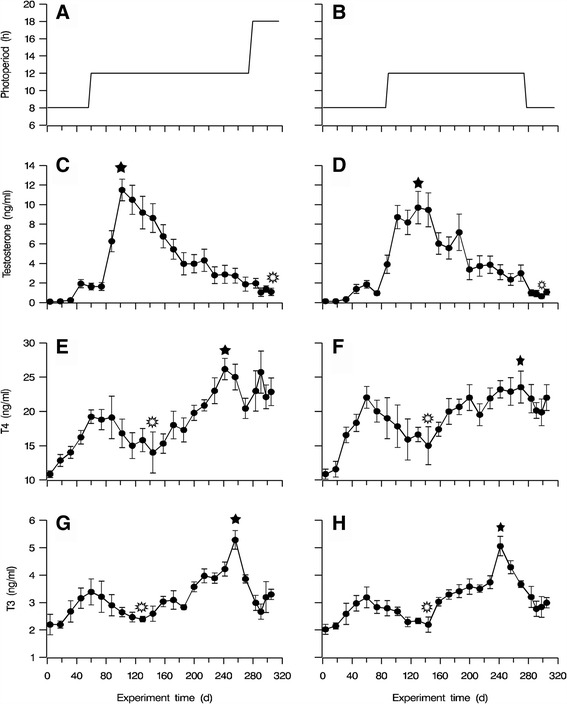
Plasma concentrations of testosterone (**c** and **d**), triiodothyronine (**e** and **f**), and thyroxine (**g** and **h**) in ganders under two artificial photoperiods. *Vertical bars* indicate standard error of the mean. The artificial photoperiod treatment (**a**) consisted of a short photoperiod of 8 h (8L:16D) for 56 days from March 1, 2015 until April 25, 2015, followed by a long photoperiod of 12 h (12L:12D) for 218 days from April 26, 2015 until November 29, 2015, and finished with a long photoperiod of 16 h (16L:8D) from November 30, 2015 until the end of the experiment, on January 6, 2016. Another artificial photoperiod treatment (**b**) consisted of a short photoperiod of 8 h (8L:16D) that lasted for 86 days from March 1, 2015 until May 29, 2015. The second phase of a 12-hour daily photoperiod (12L:12D) lasted for 184 days from May 30, 2015 until November 29, 2015. The last photophase consisted of an 8-hour (8L:16D) short photoperiod for 38 days from November 30, 2015 until January 6, 2016. The long photoperiod treatment consisted of natural illumination during the daytime plus supplementary illumination (80–100 lux) by fluorescent tubes at times after sunset and before sunrise. *Star*: high hormone concentrations; *Lace box*: low hormone concentrations

Group A ganders were initially exposed to a short photoperiod of 8 h (8L:16D) for 56 days, from March 1, 2015, to April 25, 2015 (Fig. 2a). Subsequently, the photoperiod was increased to 12 h (12L:12D) for 218 days, from April 26, 2015, to November 29, 2015 (Fig. 2a). In the final phase, the daily photoperiod was further increased to 16 h (16L:8D) for 38 days, from November 30, 2015, until the end of the experiments on January 6, 2016 (Fig. 2a). Group B ganders also underwent a three-phase photo-treatment (Fig. 2b). The first phase was the same as in group A, namely an 8-hour short photoperiod (8L:16D), but lasted for 86 days instead, from March 1, 2015, to May 29, 2015 (Fig. 2a). The second phase consisted of a 12-hour daily photoperiod (12L:12D) and lasted for 184 days, from May 30, 2015, to November 29, 2015 (Fig. 2b). Finally, from November 30, 2015, to January 6, 2016, group B ganders were exposed to an 8-hour short photoperiod (8L:16D) for 38 days (Fig. 2b). The second phase was extended to 218 days in group A and 184 days in group B, thus far exceeding the time required for progressive development of photorefractoriness under a 12L:12D cycle, which is 130–150 days, and induced considerable reproductive regression in both groups to a similar extent. Provision of the 8-hour short photoperiod was achieved by confining the ganders in the mechanically ventilated barns from 4:00 to 8:00 am, and also from 16:00 to 20:00 pm. For provision of the 12-hour photoperiod, the ganders were confined in the barns from 4:00 to 6:00 am, and also from 18:00 to 20:00 pm. The 16-hour long photoperiod treatment consisted of the natural illumination during the daytime plus the supplementary illumination of 80 to 100 lux by fluorescent tubes at times after sunset and before sunrise.

The birds were fed ad libitum with mixed feed of 12.5% crude protein, supplemented with green grass whenever possible. Feed was given during daytime, but ganders always had free access to drinking water. During the course of the experiment, blood samples were collected via wing veins into heparinized syringes every 14 days. Plasma was separated from the blood within 3 h of sample collection by centrifugation at 2000 × *g*, and stored at −20 °C until the measurements of hormone concentrations were conducted.

### Tissue collection, microscopy, and histological evaluation

On days 51, 67, 88, 131, 205, 250, and 313 of the experiment, eight ganders from each group were randomly selected and slaughtered for tissue sample collection. Immediately after collection, tissues from the hypothalamus, pituitary, and testes were snap frozen in liquid nitrogen, and stored at −80 °C until gene expression assays were performed.

A piece of testicular tissue (~0.125 cm^3^) was sliced from the left testis of each gander and was immediately fixed in 10% buffered neutral formalin solution for 24 h, and subsequently used for histology studies using an automated tissue processor (Shandon Excelsior ES, P09046, ThermoScientific, Germany). The processing involved standard step-wise dehydration with alcohol of increasing concentrations (50, 60, 95%, and absolute alcohol, respectively), clearing in xylene (two changes), infiltration, and embedding in molten paraffin wax. Tissue sections (5 μm) were mounted on glass slides and stained with hematoxylin and eosin using an automated slide stainer (Shandon VaristainGermini ES, A78000013, ThermoScientific, Germany). Stained sections were individually examined under a bright field Olympus BX63 light microscope (OLYMPUSBX63, Olympus Corporation, Tokyo) at 10× and 40× magnification for changes in the diameter of the seminiferous tubule, and the numbers of spermatogonia, spermatocytes, and elongated spermatids.

### Measurements of hormone concentrations

Plasma testosterone concentrations were determined by enzyme-linked immunosorbent assay using the Quantitative Diagnostic Kit for testosterone (North Institute of Biological Technology, Beijing, China). The assay sensitivity was 0.1 ng/mL, and the intra- and inter-assay variation coefficients were both below 15%. Serial dilutions of gander plasma samples resulted in an inhibition curve parallel to the standard curve. The r-values of the assay standard curves were greater than 0.99.

Blood concentrations of total T3 were also measured by enzyme-linked immunosorbent assay using the Quantitative Diagnostic Kit for 3,5,3′-triiodothyronine (North Institute of Biological Technology, Beijing, China). The sensitivity of the assay was 0.5 ng/mL, and the intra- and inter-assay variation coefficients were both below 10%. Similarly, blood concentrations of total T4 were measured by enzyme-linked immunosorbent assay using the Quantitative Diagnostic Kit for thyroxin (North Institute of Biological Technology, Beijing, China). The sensitivity of the assay was 0.5 ng/mL, and the intra- and inter-assay variation coefficients were both below 15%. The r-values of the assay standard curves were all greater than 0.99.

### RNA isolation, complementary DNA synthesis, and quantitative real-time polymerase chain reaction

Total RNA from hypothalamus, pituitary, and testis tissues was extracted with Trizol using a commercial kit according to the manufacturer’s instructions (RNAiso Plus, Code No. 9108, Takara, Japan). For RNA extraction, chloroform (0.2 mL) was added to the Trizol reagent (Code.no 9108, Takara, Japan), the mixture was vigorously shaken, and after 15 min, centrifuged at 12,000 × *g* for 15 min at 4 °C. Isopropanol (0.5 mL) was then added to the separated upper aqueous phase and centrifuged at 12,000 × *g* for 10 min at 4 °C. The pellet obtained was washed with 75% ethanol, air dried, and dissolved in 20 mL diethylpyrocarbonate-treated sterile water. RNA quality was assessed by electrophoresis on a 1.2% agarose gel, and the total RNA concentration was determined by measuring the absorbance at 260 nm using a spectrophotometer (NanoDrop 2000c ThermoScientific, Germany). A total of 5 μg RNA was reversely transcribed using the Takara PrimerScriptTM RT reagent kit (Perfect Real Time) (RR037, Takara, Japan) according to the manufacturer’s instructions.

Quantitative real-time polymerase chain reaction analysis was used to measure the levels of messenger RNA (mRNA) of various genes in samples from the hypothalamus, pituitary, and testes from ganders in each photoperiod phase (Fig. 2a and b). The species source and function of the genes tested are shown in Table 1. Gene-specific primers were designed using the Primer 3.0 software (www.ncbi.nlm.nih.gov/tools/primer-blast/) based on the BLAST, Ensemble, and GenBank databases (Table 2). Expression of β-actin mRNA was used as a reference gene. All polymerase chain reactions (total volume of 20 μL) consisted of 10 mL SYBR Premix Ex TaqII (Takara, Japan), 1 mL complementary DNA, 10 pmole of each forward and reverse primers (Table 2), and 7 mL ultrapure water. The thermal cycling profile used was 95 °C for 30 s, 40 cycles of 94 °C for 5 s, and 60 °C for 30 s. Fluorescence yields obtained from three replicate reactions of each complementary DNA sample were analyzed using the Mastercycler ep realplex (Eppendorf, Germany); furthermore, eight biological replicates were used to ensure the validity and accuracy of the experimental results. The relative expression levels of different genes in the tissues were calculated according to the 2^−ΔΔCT^ method [47].

**Table 1:** Species source and functions in literature of the genes tested in this study

Gene name	Species	Tissue distribution	Gene function
GnRH-I	*Anser cygnoides*	Hypothalamus	GnRH regulates the secretion of the gonadotropins LH and FSH [66].
GnIH	*Anser cygnoides*	Hypothalamus	GnIH inhibits LH secretion and reduces testis weigh [20].
VIP	*Gallus gallus*	Hypothalamus	VIP in the brain acts as a neuroendocrine factor and regulates PRL secretion [51].
TRH	*Gallus gallus*	Hypothalamus	TRH regulates thyroid stimulating hormone secretion [67].
GnRH Receptor	*Gallus gallus*	Pituitary	GnRH is a hypothalamic decapeptide that centrally controls reproduction by binding to GnRH receptors on pituitary gonadotropes and stimulating the secretion of LH and FSH [68].
GnIH Receptor	*Anser cygnoides*	Pituitary	GnIH acts directly on the pituitary via the GnIH receptor and inhibits gonadotropin release [69].
VIP Receptor-I	*Gallus gallus*	Pituitary	VIP is a hypothalamic polypeptide that controls reproduction by binding to VIP receptors on pituitary gonadotropes and stimulating PRL secretion [70].
TRH Receptor	*Gallus gallus*	Pituitary	TRH acts directly on the pituitary via the TRH receptor and controls thyroid-stimulating hormone (TSH) secretion e [71].
FSH beta	*Anser cygnoides*	Pituitary	FSH stimulates gonadal growth and estrogen secretion by Sertoli cells.
LH beta	*Anser cygnoides*	Pituitary	LH controls estrogen and androgen production by mature ovarian follicles, and regulates androgen production by Leydig cells [72].
PRL	*Anser cygnoides*	Pituitary	PRL inhibits gene expression of steroidogenic enzymes and reduces testis weight [73, 74].
TSH beta	*Anser cygnoides*	Pituitary	TSH is a glycoprotein released from the adenohypophysis that activates iodine uptake, thyroid hormone synthesis, and the release of thyroid hormones from the thyroid gland [75].
LH Receptor	*Anser cygnoides*	Testis	LH receptor is one of the three glycoprotein hormone receptors that is necessary for critical reproductive processes, including gonadal steroidogenesis, oocyte maturation and ovulation, and male sex differentiation [76].
FSH Receptor	*Anser cygnoides*	Testis	FSHR is a transmembrane receptor that interacts with FSH, and its activation is necessary for the hormonal functions of FSH [77].
3-beta HSD	*Anser cygnoides*	Testis	3-beta HSD catalyzes an obligatory step in the biosynthesis of all classes of hormonal steroids, namely, the oxidation/isomerization of 3-beta-hydroxy-5-ene steroids into the corresponding 3-keto-4-ene steroids in gonadal as well as in peripheral tissue [78].
StAR	*Anser cygnoides*	Testis	StAR plays a critical role in steroid hormone synthesis, and it is thought to increase the delivery of cholesterol to the inner mitochondrial membrane where P450scc resides [79].

Abbreviations: *GnRH* gonadotropin-releasing hormone, *GnIH* gonadotropin-inhibitory hormone, *VIP* vasoactive intestinal peptide, *TRH* thyrotropin releasing hormone, *FSH* follicle-stimulating hormone, *LH* luteinizing hormone, *PRL* prolactin, *TSH* thyroid-stimulating hormone, *3-beta HSD* three beta-hydroxysteroid dehydrogenase, *StAR* steroidogenic acute regulatory protein

**Table 2:** Primers used in the real-time quantitative PCR assay of gene transcription

Gene name	Accession number	Primer sequences (5′-3′)	Annealing temperature (°C)	PCR product (bp)
β-actin	L08165.1	upstream: TGACGCAGATCATGTTTGAGA	60	159
		downstream: GCAGAGCGTAGCCCTCATAG		
GnRH-I	EF495207.1	upstream: CTGGGACCCTTGCTGTTTTG	60	232
		downstream: AGGGGACTTCCAACCATCAC		
GnIH	KC514473.1	upstream: ATCTACCTAGGCATGCTCCAA	58	115
		downstream: ACAGGCAGTGACTTCCCAAAT		
VIP	DQ023159	upstream: ACCAGTGTCTACAGCCATCTTTTG	58	204
		downstream: AGGTGGCTCAGCAGTTCATCTACA		
TRH	NM_001030383.2	upstream: GCAAGAGGGGCTGGAATGAT	58	133
		downstream: ATGGCAGACTGCTGAAGGTC		
GnRH Receptor	KJ659046.1	upstream: TCTGCTGGACCCCCTACTAC	60	127
		downstream: TCCAGGCAGGCATTGAAGAG		
GnIH Receptor	KF696709.1	upstream: GTCGTCATGTACACCCGCAT	56	103
		downstream: TCTTGCGAGACACCTTCCTC		
VIP Receptor-I	NM_001097523.1	upstream: TACTGCGTCATGGCCAACTT	58	153
		downstream: TGTCCAAGCGGTGATGAACA		
TRH Receptor	NM_204930.1	upstream: CTATGGCTATGTGGGGTGCC	60	189
		downstream: ACTGAGGCGAAAGACCAGAC		
FSH beta	EU252532	upstream: GTGGTGCTCAGGATACTGCTTCA	60	209
		downstream: GTGCAGTTCAGTGCTATCAGTGTCA		
LH beta	DQ023159	upstream: GACCCGGGAACCGGTGTA	58	90
		downstream: AGCAGCCACCGCTCGTAG		
PRL	DQ023160	upstream: TGCTCAGGGTCGGGGTTTCA	56	218
		downstream: GCTTGGAGTCCTCATCGGCAAGTT		
TSH beta	FJ797681.1	upstream: CTCTGTCCCAAAACGTGTGC	56	121
		downstream: CCACACTTGCAGCTTATGGC		
LH Receptor	XM_013192443.1	upstream: CGGATACACAACGATGCCCT	60	74
		downstream: GACTCCAGTGCCGTTGAAGA		
FSH Receptor	KC477215.1	upstream: CCTAGCCATTGCTGTGCATTT	60	106
		downstream: TGCCAGGTTGCTCATCAAGG		
3-beta HSD	KC310447.1	upstream: TGTGACGTTCCTGTACCGTG		153
		downstream: TTACAACGGGTACACGCCTC		
StAR	KF958133.1	upstream: GGAGCAGATGGGAGACTGGA	56	177
		downstream: CGCCTTCTCGTGGGTGAT		

### Statistical analysis

**Fig. 3:**
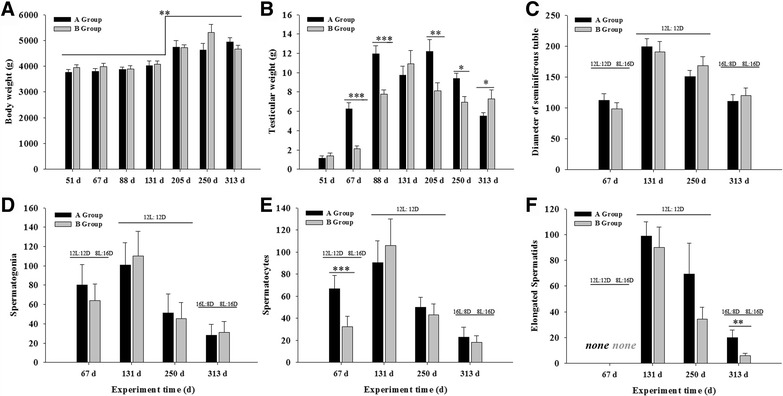
Body (**a**) and testicular weights (**b**)﻿, ﻿and testicular histological data (**c**-**f**) of Yangzhou ganders maintained under two artificial photoperiods. Changes in the diameter of the seminiferous tubules (**c**), the number of spermatogonia (**d**), spermatocytes (**e**), and elongated spermatids (**f**) were measured within 8 individial seminiferous tubules. Each bar represents the mean value of six determinations including the standard error. *, ** and *** indicate statistical significance based on *P* < 0.05, *P* < 0.01 and *P* < 0.001, respectively

**Fig. 4:**
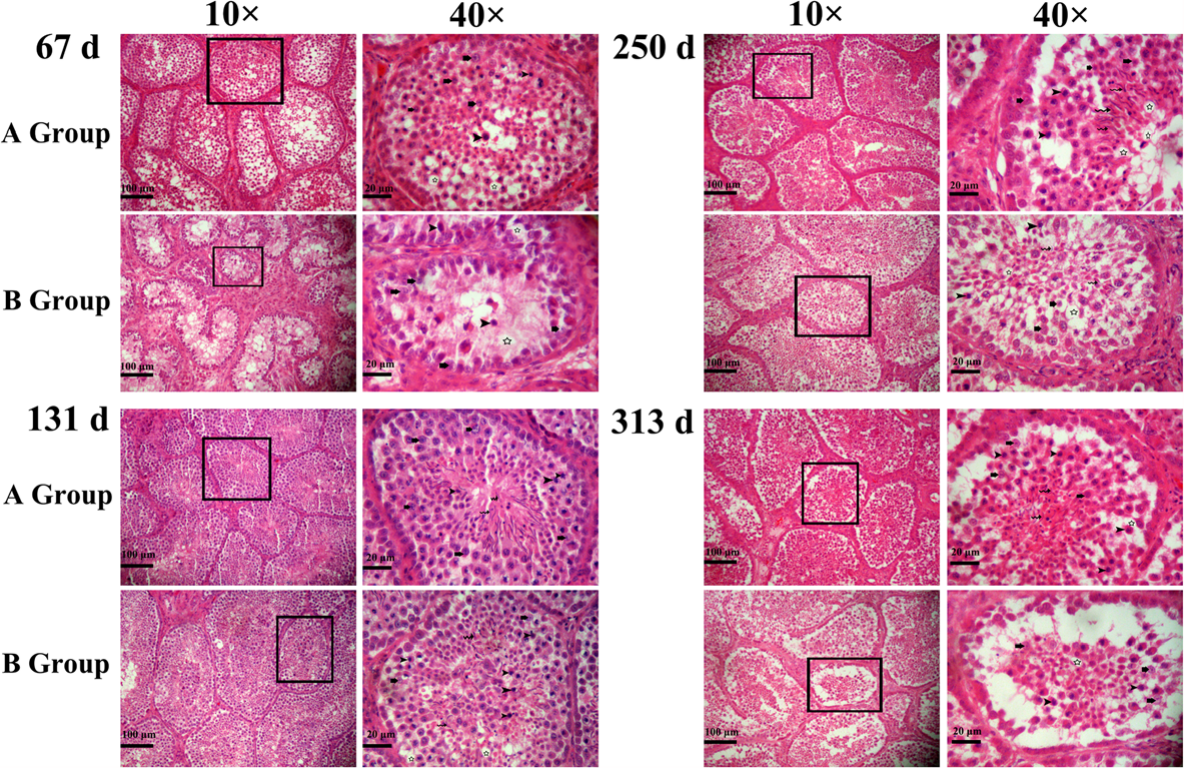
Histological analysis of sections from Yangzhou goose gander testis. Sections from Yangzhou goose gander were collected on days 67, 131, 250, and 313 and were stained with hematoxylin and eosin *Black arrowhead*: spermatogonia; *black arrow*: primary spermatocyte; *black arrow with tail*: elongated spermatid; *star*: Sertoli cell vacuolation. *Scale bar* represents 100 μm in 10 × and 20 μm in 40 ×

**Fig. 5:**
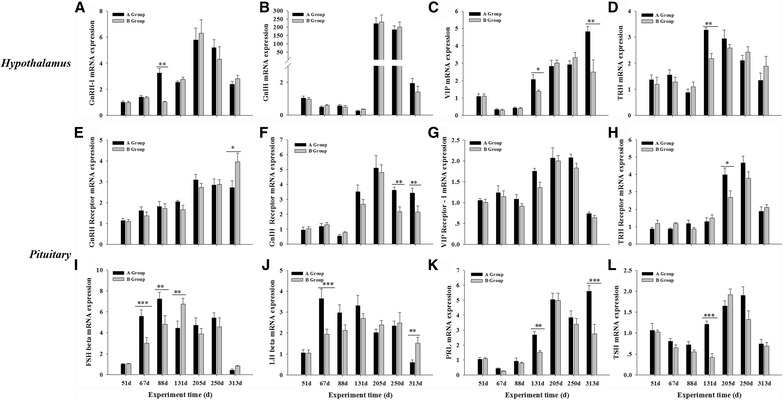
Hypothalamic (**a**-**d**) and pituitary (**e**-**l**) mRNA levels in Yangzhou goose ganders under two artificial photoperiods. Graphs **a** to **d** repre﻿sent mRNA expression levels of hypothalamic releasing hormones GnRH-I, GnIH, VIP and TRH, respectively, while **e** to **h** their corresponding receptors in the pituitary gland, and **i** to **l** the FSH, LH, PRL and TSH pituitary hormones or beta subunits wherever applicable. ﻿Each value represents the average of data from eight independent culture experiments. Data are shown as mean values ± standard error of the mean. *, ** and *** indicate statistical significance based on *P* < 0.05, *P* < 0.01and *P* < 0.001, respectively, between the treatments

**Fig. 6:**
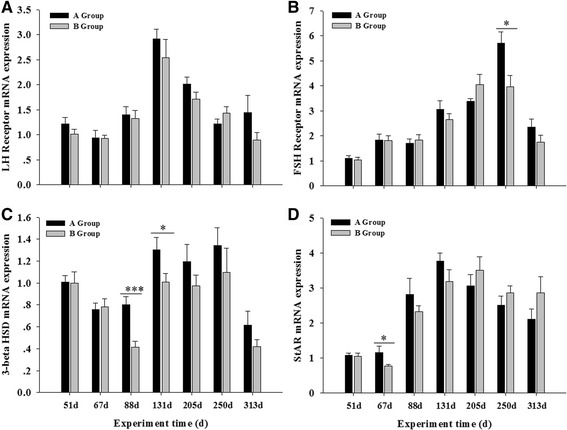
Testicular mRNA levels ﻿of LH receptor (**a**), FSH receptor (**b**), 3-beta HSD (**c**) and StAR (**d**) in Yangzhou goose ganders under two artificial photoperiods. Each value represents the average of data from eight independent culture experiments. Data are shown as mean values ± standard error of the mean. *, ** and *** indicate statistical significance based on *P* < 0.05, *P* < 0.01and *P* < 0.001, respectively. between the treatments

The results were mean values ± standard error of 12 (Fig. 2) and 8 (Figs. 3, 4, 5 and 6) replicate samples in each treatment groups. Differences of plasma concentrations of testosterone, T3, and T4 between photo-programs were analyzed with two-way analyses of variance (two-way ANOVA). Gene expression levels were analyzed with one-way analyses of variance (one-way ANOVA) with main treatments of the two photo-programs and the serial sampling of the experiment. Differences of each effect means were compared by the mean ± standard error of mean and considered significant at *P* < 0.05. Statistical analyses were performed using IBM SPSS software (ver. 11.0; IBM SPSS, Armonk, NY, USA).

## Results

### Concentrations of plasma testosterone

In the first half of the 56-day 8-hour short photoperiod phase, plasma testosterone concentrations were low, below 0.5 ng/mL, in ganders from both groups, A and B (Fig. 2c–d). With the continuation of the 8-hour photoperiod, the concentrations were slightly elevated to approximately 2 ng/mL, and at the end of the 8-hour photoperiod phase, on day 86 of the experiment, to 4 ng/mL in group B (Fig. 2d). At the same time, in group A ganders, which were already exposed to a 12-hour photoperiod since one month, plasma testosterone concentrations had already increased to significantly higher levels of approximately 6 ng/mL (*P* = 0.024, *F* = 10.743) (Fig. 2c). Subsequently, as both gander groups continued to be exposed to the second 12-hour photoperiod phase, plasma testosterone concentrations continued to increase rapidly, and they reached peak levels (8–11 ng/mL) from day 100 to day 140 of the experiment. Toward the end of the 12-hour photoperiod phase or on day 274 of the experiment, testosterone concentrations in both groups progressively decreased to lower levels, approximately 4 ng/mL. Thereafter, in the third photoperiod phase (days 275–313 of the experiment) plasma testosterone concentrations further decreased to levels below 2 ng/mL in both groups (Fig. 2c–d), despite their divergent treatments.

### Concentrations of plasma T4 and T3

The general pattern of the concentrations of plasma T4 and T3, and that of their relative ratio (T4/T3), was reciprocal to that of testosterone concentrations. For example, in the first short 8-hour photoperiod phase, both plasma T4 and T3 concentrations increased from their initial low levels and peaked on day 60 of the experiment. Similarly, the values of the T4/T3 ratio (5.86 and 7.07 for groups A and B respectively) were significantly higher than those at the beginning of the experiment (*P* = 0.031, *F* = 20.121 and *P* = 0.047, *F* = 25.339, for groups A and B respectively). The concentrations of both hormones started then to decrease, reaching a low plateau value at around day 140 of the experiment (2–3 months into the 12-hour photoperiod phase), but they subsequently increased, reaching peak levels around days 240 to 260 of the experiment, or almost the end of the 12-hour photoperiod phase, especially for T3. Until day 270, the values of the T4/T3 ratio were maintained at relatively high levels: 9.5611 and 7.27 for groups A and B, respectively. Then they started to decrease again, especially for T3, but displayed a small increase during the final phase of photoperiod change, around days 297 to 313 of the experiment (Fig. 2f). Throughout the experiment, the changing patterns of T4 and T3 concentrations were highly similar in both groups A and B.

### Body weight, testis morphometry, and histology

In both gander groups, body weight exhibited a two-phase variation (Fig. 3a). Before day 131 of the experiment, the weight of live ganders was maintained around 3.95 kg, but toward the end of the experiment on day 313, it increased to significantly higher values, in the range of 4.73–4.93 kg (*P* = 0.005, *F* = 16.215), for both gander groups (Fig. 3a).

The weight of the left testis in both groups was below 2 g on day 51, during the 8-hour photoperiod (Fig. 3b). For the ganders of group B, it remained the same until day 67 of the experiment. In the ganders of group A however, the increase in the photoperiod from 8 to 12 h for just 12 days promptly stimulated testicular growth, and the weight of the left testis increased to approximately 6 g (Fig. 3b), and continued to increase until the day 88 of the experiment, when it reached a peak value significantly higher than that observed in the ganders of group B (*P* = 0.000, *F* = 17.952). In the latter group, the peak weight of 11 g was reached only on day 131 of the experiment (Fig. 3b). Thereafter, testicular weight decreased in both groups. However, at the end of the experiment (day 313) the testicular weight of ganders in group A was significantly lower than that of ganders in group B, when the two groups were exposed to photoperiods of 16 and 8 h, respectively (Fig. 3b).

Microscopic observation of testis histological sections (Fig. 4) revealed that for both gander groups, the seminiferous tubule diameter (Fig. 3c) and the number of spermatogonia (Fig. 3d), spermatocytes (Fig. 3e), and elongated spermatids (Fig. 3f) changed in a similar fashion throughout the experiment: they increased from low values on day 67, to peak values on day 131, and decreased thereafter to low values again until the end of experiment (Fig. 4). It should be noted that on day 67, the spermatocyte number (Fig. 3e) was significantly higher in group A, which was already exposed to a 12-hour photoperiod, than in group B, which was still exposed to an 8-hour photoperiod (*P* = 0.000, *F* = 24.965), as was also the case with the seminiferous tubule diameter (Fig. 3c). On day 313, when the experiment ended, there were still more elongated spermatocytes in group A than in group B (Fig. 4 and Fig. 3f). In addition to the changes in diameter, the number of vacuoles in the seminiferous tubules was higher both at the beginning and end of the experiment than at the peak of the reproductive activity on day 131, where the seminiferous tubules were more compact and in a cell-filled state.

### Gene expression levels

#### Hypothalamus and pituitary genes

All of the 16 genes tested exhibited photoperiod-dependent mRNA level regulation throughout the experiment (Fig. 5). The hypothalamic GnRH-I mRNA levels changed similarly in ganders from groups A and B (Fig. 5a): they increased from the beginning of the experiment to reach a peak level on day 205, during the 12-hour photoperiod, and then steadily decreased, with an exception on day 88, when they were significantly higher in group A than in group B. Similarly, GnIH gene transcription levels varied in a strikingly similar manner in both groups (Fig. 5b): they steadily decreased from their already low levels at the beginning of the experiment to a minimum value on day 131, during the 12-hour photoperiod, and then remarkably increased by 200-fold to a maximum from day 205 to day 250, before they finally decreased again. The changes in VIP and TRH gene transcription in the hypothalamus exhibited changes similar to that of GnIH throughout the experiment (Fig. 5c–d): their mRNA levels decreased to or remained at low levels until day 88 of the experiment, and then increased to higher levels on day 131, during peak reproductive activities. However, on two occasions, namely on days 131 and 313 of the experiment, the VIP mRNA levels were significantly higher in group A than in group B (*P* = 0.013, *F* = 13.542 and *P* = 0.002, *F* = 20.189), while the same was true for the TRH mRNA levels on day 131.

In the pituitary gland, the GnRH receptor mRNA levels increased steadily from day 51, during the short 8-hour photoperiod, to a plateau value at day 250 in group A, whereas in group B, they further increased to a peak value at the end of the experiment (Fig. 5e). The GnIH receptor mRNA levels (Fig. 5f) remained low before day 88, but they started to rise from day 131, and reached a peak level on day 205, then decreased in both groups. However, on days 250 and 313, the post-peak mRNA levels were higher in group A than in group B (*P* = 0.001, *F* = 24.773 and *P* = 0.004, *F* = 7.200 for days 250 and 313 respectively) (Fig. 5f). Throughout the experiment, the mRNA levels of the VIP receptor changed in a manner similar to that of the GnIH receptor, as did the TRH receptor mRNA levels (Fig. 5g–h).

FSH beta mRNA (Fig. 5i) expression levels were initially low under the 8-hour photoperiod, and gradually increased to the peak on day 131, and then decreased to the nadir on day 313 in Group B ganders. The FSH mRNA levels on days 67 and 88 were significantly higher in ganders of group A than in those of group B (*P* = 0.000, *F* = 11.710 and *P* = 0.005, *F* = 6.744 for days 67 and 88 respectively). Similarly, in group A, the LH beta mRNA levels (Fig. 5j) increased from their low levels during the 8-hour photoperiod on day 51 to peak levels on day 67, just 12 days after the photoperiod was increased from 8 to 12 h. This peak was maintained until day 131 and then gradually decreased to a minimum on day 313 (end of the experiment). In addition, on day 67, they were significantly higher in group A than in group B (*P* = 0.000, *F* = 25.221), whose LH beta transcription patterns changed similarly to those of FSH beta. The PRL mRNA levels (Fig. 5k) remained low before day 88 in both groups, then started to increase from day 131 to a peak level on day 205, and thereafter decreased. In group A, on days 131 and 313, PRL transcription was significantly higher than in group B (*P* = 0.002, *F* = 12.867 and *P* = 0.000, *F* = 21.107 for days 131 and 313 respectively). The levels of TSH beta mRNA (Fig. 5l) changed in both groups in a manner similar to that of GnIH, except on day 131, where the expression was abruptly and significantly higher in group A than in group B (*P* = 0.000, *F* = 20.946).

#### Testicular genes

The FSH receptor mRNA levels (Fig. 6a) increased steadily from the beginning of the experiment; they reached peak levels during days 205–250, and then decreased to low levels at end of experiment. In both groups of ganders, the LH receptor mRNA levels (Fig. 6b) also increased, but they peaked on day 131, and then decreased until the end of experiment. This changing pattern was also true for the StAR mRNA levels (Fig. 6c), except that on day 67 they were significantly higher in group A than in group B (*P* = 0.012, *F* = 5.480). The mRNA levels of 3-beta hydroxysteroid dehydrogenase remained low or decreased before day 88, and then increased to peak levels from day 131 to 250, and finally decreased again to low levels at the end of experiment (Fig. 6d). On days 88 and 131, the levels were also higher in group A than in group B (*P* = 0.000, *F* = 16.329 and *P* = 0.037, *F* = 3.879 for days 88 and 131 respectively).

## Discussion

This study systematically investigated the molecular mechanisms associated with testicular development and regression in response to artificial photoprograms in Yangzhou goose ganders. A wide spectrum of variables was assessed, including testis histology, hormone plasma concentrations, and gene transcription patterns. In addition to the general responses to an increase in photoperiod (i.e. testicular development and subsequent regression), some minor responses in the timing of testicular development and gene transcription were also observed in relation to subtle changes in photoperiod. These results reinforce the proposition that the Yangzhou goose is a long-day breeding bird, and provide new insights into the molecular mechanisms that mediate the photoperiod-regulated seasonal changes in reproductive activities.

It was also noted that after 40 days of exposure to an 8-hour short photoperiod, testosterone concentrations slightly increased in both gander groups studied (A and B). This phenomenon may be linked to the weak expression of gonadal or reproductive activities during short days after dissipation of refractoriness to the long days [4, 45, 48]. In addition, in group B and on day 88 of the experiment, refractoriness to a short photoperiod also led to spontaneous testicular development immediately before the photoperiod was increased. Subsequently, an increase in daily photoperiod from 8 to 12 h resulted in rapid growth of the testes, accompanied by an increase in testicular weight from less than 2 g during the 8-hour short photoperiod to approximately 6 g in just 10 days, and to 12 g in 21 days. The delay of 28 days in photoperiod increase for group B with respect to that of group A postponed testicular growth at a similar pace. Following 40 days of exposure to a 12-hour photoperiod, the ganders reached peak reproductive activities as represented by the high levels of plasma testosterone and active spermatogenesis in the testis at days 100–150 of the experiment. After that time point, refractoriness to the long photoperiod gradually commenced, as reflected by a decrease in testicular weight, disappearance of spermatozoa in the seminiferous tubules of the testes, and especially by the steady decrease in the concentration of plasma testosterone. The 12-hour equatorial photoperiod was adopted in this study not only to induce the reproductive activities of ganders, but also to test how photorefractoriness develops in the Yangzhou goose. Clearly, ganders develop a kind of “absolute photorefractoriness” similar to that reported in starlings [49], in contrast to the “relative photorefractoriness” observed in quails [49] and sheep [50]. Therefore, at the end of the experiment, the testosterone concentration decreased to minimal levels in both groups A and B. Such changes should be the result of different mechanisms, i.e. the further refractoriness to long photoperiod in group A by an increase in photoperiod to 16 h (as was indicated by upregulation of VIP and PRL mRNA levels), and the inhibition of reproductive activity in group B by a decrease in photoperiod.

It has been proposed that the endocrine mechanism mediating the photoperiod-dependent regulation of seasonal reproduction is through the coordinated production and secretion of the gonadotrophins LH and PRL by the pituitary gland [14]. Increases in photoperiod first drive LH secretion, which stimulates gonadal development and initiates the breeding season, and then PRL secretion, which initiates the development of photorefractoriness, and after reaching peak levels, reduces LH secretion and leads to gonad regression. Since production and secretion of LH is regulated by the hypothalamic-releasing hormones GnRH and GnIH [13, 21, 25], and that of PRL by VIP [32, 51], these hormones must be involved in the photoperiod-dependent regulation of seasonal breeding [3, 13, 14]. Therefore, in the present study, the mRNA levels of GnRH, GnIH, and VIP were measured in the hypothalamus of the ganders, and those of their receptors in the pituitary gland. In both groups of ganders, initiation of reproductive activities after prolonged exposure to a short 8-hour photoperiod, or a state of refractoriness to the short photoperiod, concomitantly occurred with a steady decrease in the mRNA levels of VIP and GnIH. Switching from an 8-hour to a 12-hour photoperiod upregulated the transcription of hypothalamic GnRH, but further downregulated that of GnIH, which reached a minimum when ganders were in a fully active reproductive state. Changes in GnRH/GnIH gene transcription in response to an increase in photoperiod were accordingly reflected by changes in FSH and LH mRNA levels in the pituitary gland. It must be noted that the earlier exposure to a 12-hour long photoperiod in group A was associated with an earlier upregulation of both FSH and LH mRNA levels. This could explain the earlier testicular growth and rise in the concentrations of plasma testosterone observed in the ganders of group A.

Notwithstanding the above observations, the post-peak decrease in reproductive activities after a prolonged exposure to a 12-hour long photoperiod (day 205 of the experiment) was associated with a greater than 300-fold upregulation in the mRNA levels of GnIH. This increase in GnIH gene transcription and hence secretion of the hormone may be the driving force in inducing reproductive regression during the development of refractoriness, despite a 2- to 3-fold increase in GnRH mRNA levels after continued exposure to a long photoperiod. The temporal elevation of GnRH mRNA levels from day 205 to day 250 may not be photoperiod-driven, but could rather be caused by a decreased negative control feedback arising from the diminished plasma testosterone concentrations. Whereas the declined GnRH expression at end of experiment could be the true effect of photoperiodic, by the refractoriness under 16 h photoperiod or by the inhibition under 8 h photoperiod. On the other hand, the inhibition by GnIH of pituitary gonadotrophin synthesis could be mediated via two pathways, one by the direct effect on pituitary gland and the other indirectly via reducing GnRH secretion by inhibiting GnRHneurons in the hypothalamus [13]. The inhibitory effect of GnIH on gonadotrophin expression and secretion could be at a maximum level starting from day 205 of the experiment, that is approximately 150 days after switching to the long 12-hour photoperiod, if pituitary GnIH receptor mRNA levels were also included. Another factor that is important for the development of refractoriness to a long photoperiod is PRL, whose mRNA levels reached their highest value on day 205 of the experiment, when the mRNA levels of the GnIH/GnIH receptor also reached their highest value and could exert their maximal inhibitory impact on gonadotrophin secretion. The mRNA levels of VIP and the VIP receptor, which stimulate pituitary PRL secretion, were already upregulated from day 131 of the experiment. As ganders in group A were exposed to a 12-hour photoperiod one month earlier than ganders in group B, the VIP/VIP receptor mRNA levels were also observed to rise earlier in the former group. Furthermore, at the end of the experiment on day 313, both VIP and PRL mRNA levels were further upregulated in group A, in response to an increase in photoperiod from 12 h to 16 h 35 days earlier than in group B. Such an upregulation did not occur in the ganders of group B, which experienced a decrease of photoperiod from 12 h to 8 h.

The photostimulation and refractoriness of reproductive activities were also analyzed in terms of testicular steroidogenesis gene transcription patterns. The transcription of LHR, which mediates the gonadotrophic effects of LH, displayed a typical rise-and-fall pattern, following that of the changes in testicular weight and plasma testosterone concentration. A similar effect was observed for StAR and 3-beta hydroxysteroid dehydrogenase transcription. Thus, toward the end of the experiment, when both LH beta and LH receptor mRNA levels significantly subsided, so did the mRNA levels of the steroidogenic genes StAR and 3-beta hydroxysteroid dehydrogenase. Depletion of these key enzymes of steroidogenesis would result in diminished testosterone production, as shown by the steady decline of plasma testosterone concentration toward the end of the experiment. This, in turn, would impair spermatogenesis, resulting in testis atrophy and reduced testis weight. Of the testicular genes tested, the FSH receptor mRNA levels peaked during days 205–250 of the experiment; that is, during the testicular regression process. This illustrates that the biological role of FSH in the regulation of testicular functions may occur during the early stages of spermatogenesis (i.e., germ cell mitosis before the spermatocyte stage) [52]. Testosterone is more important during the later stages of spermatogenesis, when morphologic changes lead to spermatid formation. As a result, on day 313 of the experiment, when testosterone and FSH levels were minimal, no spermatids were observed in the testes of ganders in either group.

The thyroid hormones function as molecular switches in the regulation of seasonal reproduction [11, 53], and are involved in both photoperiod-induced reproductive stimulation and refractoriness. In Japanese quail, removal of thyroid hormones by thyroidectomy reduces the maximal extent of testis development induced by a long photoperiod [49] In the quail, it was discovered that TSH is synthesized in the pars tuberalis of the pituitary gland in response to an increase in photoperiod, and through retrograde uptake, it stimulates the ependymal cells in MBH to express type 2 iodothyroninedeiodinase, which converts T4 to T3 [41, 54]. Such locally produced T3, stimulates the release of GnRH into the portal vascular system by inducing morphological or plasticity changes in GnRH-secreting neurons [41], resulting in elevated LH secretion and full reproductive activities [11, 13]. On the other hand, thyroid hormones have a permissive role in the development of photorefractoriness that leads to reproductive regression in both mammals and birds [34, 39, 55–57]. However, when there is a complete lack of thyroid hormones, reproductive activities still change in response to daily photoperiod in both thyroidectomized birds [49] and red deer [34]. Therefore, it appears that the photoperiod-dependent regulation of seasonal reproduction or a GnRH pulse generator should still be operating at brain centers higher than MBH, while local T3 production in the MBH might amplify or modify this central effect. It is possible that systemic effects, in contrast to the above-discussed local actions of thyroid hormones in the MBH, may mediate reproductive inhibiting actions. In several animal species living in temperate zones, such as ducks [36], Moscovy drakes [37] and geese [38], there was a seasonal rise of plasma thyroid hormones around the time of reproductive regression. In domestic ganders, it has been shown that the concentrations of T4 and T3 decreased rapidly with increasing testosterone concentration, and with the approach of the non-breeding season, testosterone concentration decreased rapidly, whereas T4 concentration showed the opposite trend [38]. In the present study, the changes in plasma concentrations of both T4 and T3 exhibited a pattern opposite to that of testosterone. The seasonal fluctuations of thyroid hormones might be an aspect of the general seasonal physiological changes; alternatively, high thyroid hormone levels during the non-breeding season might facilitate, if not altogether cause, the termination of the breeding season, and facilitate the manifestation of other seasonal events such as growth [34], as discussed further below. It should also be noted that the involvement of thyroid hormones in the refractoriness to a 12-hour long photoperiod, observed in this study, occurred with a concomitant upregulation of the TRH, TRH receptor, and TSH mRNA levels, as well as of the VIP, VIP receptor, and PRL mRNA levels. Indeed, in European starlings, abolishing the development of photorefractoriness by thyroidectomy also prevented the corresponding increase in PRL secretion [39].

Thyroid hormones, PRL, and GnIH not only stimulate the development of photorefractoriness that leads to reproductive regression, but can also regulate feed intake and growth. GnIH has been shown to stimulate feed intake in both animals [58] and fowls [59, 60]. Based on this, it was proposed that may be a molecular switch between feeding, and hence somatic growth, and reproduction [58]. Furthermore, both thyroid hormones and PRL are essential for long photoperiod-induced seasonal increases in feed intake and live weight gain simultaneously to reproductive regression [34, 61], whereas PRL has been found to be orexigenic in domestic cows [62, 63]. It appears that thyroid hormones and PRL also fulfill the roles of molecular switches between growth and reproduction. Consequently, at the late stages of the experiment, the reproductive system regression accompanied by a significant weight increase in ganders could well be the result of actions of these three molecular switches. It was proposed that the seasonal somatic growth in wild animals that occur at the same time of reproductive regression [34, 64, 65] promotes animal survival throughout the hardships of winter. It appears that this strategy remains operative in the domestic goose as well, and involves the same endocrine regulatory factors.

## Conclusions

In summary, reproductive stimulation of long-day breeding Yangzhou goose ganders was characterized by GnRH upregulation and GnIH and VIP downregulation in the hypothalamus during long or increasing photoperiods. This, in turn, stimulated the transcription of the gonadotrophin gene, whereas inhibited that of the PRL gene in the pituitary gland, which is known to stimulate testicular development by upregulating genes important for steroidogenesis and spermatogenesis. On the other hand, development of photorefractoriness was characterized by hyper-regulation of GnIH transcription in the hypothalamus, in addition to transcription upregulation of VIP, TRH, and their receptors in the pituitary gland. Consequently, gonadotrophin transcription was downregulated, whereas that of PRL and TSH was upregulated. The combined effects of these changes resulted in reproductive system regression.
